# The effects of vigorous intensity exercise in the third trimester of pregnancy: a systematic review and meta-analysis

**DOI:** 10.1186/s12884-019-2441-1

**Published:** 2019-08-07

**Authors:** Kassia S. Beetham, Courtney Giles, Michael Noetel, Vicki Clifton, Jacqueline C. Jones, Geraldine Naughton

**Affiliations:** 1 School of Behavioural and Health Sciences, Australian Catholic University, 1100 Nudgee Road, Banyo, Brisbane, Queensland 4014 Australia; 2 Pregnancy and Development, Mater Research Institute-University of Queensland, Translational Research Institute, South Brisbane, Queensland Australia; 3 Obstetrics and Gynaecology Department, Royal North Shore Hospital, Sydney, New South Wales Australia; 4 Department of Educational Studies, Macquarie University, Sydney, New South Wales Australia; 5 School of Health Sciences, Swinburne University of Technology, Melbourne, Victoria Australia

**Keywords:** High intensity, Physical activity, Gestation, Prenatal, Antenatal, Intrauterine growth restriction, Small for gestational age, Maternal weight gain, Infant

## Abstract

**Background:**

Fetal growth is dependent upon utero-placental vascular supply of oxygen and nutrients from the mother and has been proposed to be compromised by vigorous intensity exercise in the third trimester. The aim of this systematic review was to investigate the effects of vigorous intensity exercise performed throughout pregnancy, on infant and maternal outcomes.

**Methods:**

Electronic searching of the PubMed, Medline, EMBASE, Cochrane Library, Web of Science and CINAHL databases was used to conduct the search up to November 2018. Study designs included in the systematic review were randomised control trials, quasi-experimental studies, cohort studies and case-control studies. The studies were required to include an intervention or report of pregnant women performing vigorous exercise during gestation, with a comparator group of either lower intensity exercise or standard care.

**Results:**

Ten cohort studies (*n* = 32,080) and five randomized control trials (*n* = 623) were included in the systematic review (*n* = 15), with 13 studies included in the meta-analysis. No significant difference existed in birthweight for infants of mothers who engaged in vigorous physical activity and those who lacked this exposure (mean difference = 8.06 g, *n* = 8006). Moreover, no significant increase existed in risk of small for gestational age (risk ratio = 0.15, *n* = 4504), risk of low birth weight (< 2500 g) (risk ratio = 0.44, *n* = 2454) or maternal weight gain (mean difference = − 0.46 kg, *n* = 1834). Women who engaged in vigorous physical activity had a small but significant increase in length of gestational age before delivery (mean difference = 0.21 weeks, *n* = 4281) and a small but significantly reduced risk of prematurity (risk ratio = − 0.20, *n* = 3025).

**Conclusions:**

Findings from this meta-analysis indicate that vigorous intensity exercise completed into the third trimester appears to be safe for most healthy pregnancies. Further research is needed on the effects of vigorous intensity exercise in the first and second trimester, and of exercise intensity exceeding 90% of maximum heart rate.

**Trial registration:**

PROSPERO trial registration CRD42018102109.

**Electronic supplementary material:**

The online version of this article (10.1186/s12884-019-2441-1) contains supplementary material, which is available to authorized users.

## Background

Moderate intensity aerobic exercise throughout pregnancy is known to result in lower caesarean delivery rates, lower incidence of gestational diabetes and hypertensive disorders, decreased maternal weight gain, and improvements in antenatal and postnatal depression, and has not been found to negatively affect birth weight [1–3]. However, studies investigating the effects of vigorous intensity exercise on birth weight have been mixed [4–8]. This is clinically important as birth weight is the single most important predictor of neonatal morbidity and mortality [9]. Research has shown that fetal hypoglycemia in hypoxic conditions can result in infants born small for gestational age [10]. So while moderate intensity exercise throughout pregnancy is beneficial, it is not known whether vigorous intensity exercise is detrimental, particularly in the third trimester when the needs of the fetus are greater.

Decreases in utero-placental blood flow occurs during vigorous intensity exercise, and has been shown to result in fetal bradycardia [11]. Physical exertion demands greater substrate utilisation, and as such re-directs blood to the working muscles, whilst also generating heat and excess by-products [12]. The combination of these adaptations challenges the greater demands required by the fetus during pregnancy. Indeed, reduced fetal movement after vigorous intensity exercise in the third trimester has been shown in studies with both conditioned and unconditioned mothers [13]. It seems likely that the increasing physiological demands during each trimester of pregnancy require variation in exercise training accordingly. However, current guidelines for pregnancy are not trimester-specific.

The American College of Sports Medicine (ACSM) recommend moderate intensity exercise throughout pregnancy; however, the guidelines around vigorous intensity exercise are not as clear [14]. Vigorous and high-intensity exercise is defined as being at least 70% of maximum heart rate (MHR), or an activity in which a conversation generally cannot be maintained [15]. There is limited participation in vigorous intensity exercise in pregnancy [16], perhaps due to the fact it requires a significant increase in workload of greater than 6–9 times resting levels of metabolism (6–9 METs) [15]. This is thought to be due to an increase in discomfort in progressing pregnancy [16], or, potentially is indicative of a lack of consensus around the safety of participating in this type of training. The threshold for achieving vigorous intensity exercise during pregnancy is considered to be lower than the non-pregnant population due to autonomic nervous system modulation and subsequent attenuation of maximum heart rate and elevation of resting heart rate [17]. Indeed, if women are trying to achieve vigorous intensity workload based on an aerobic capacity of 60–80% heart rate reserve or VO_2_peak, it is recommended by the Canadian Guideline for Physical Activity throughout Pregnancy that women target a heart rate of between 142 and 169 bpm, depending on their age [18]. This range is lower than the target heart rate of non-pregnant populations. The haemodynamic variances throughout the stages of pregnancy, which are also dependent on age and fitness, provides insight to the lack of guidance behind vigorous intensity exercise prescription in pregnant populations. Moreover, it highlights the need for a subjective measure of intensity to be used concurrent to any objective measure [17]. It is currently recommended that if patients are completing vigorous intensity exercise before pregnancy they should be able to continue throughout pregnancy, but with caution. This is also the recommendation by The Royal Australian and New Zealand College of Obstetricians and Gynaecologists [19].

The current understanding of the benefits of vigorous intensity exercise during pregnancy lie predominantly in decreased maternal weight gain. Moreover, it has been suggested that vigorous intensity exercise is an important goal for pregnant women, especially among the overweight or obese, previously inactive, or those with gestational diabetes [20, 21]. A study by Clapp, et al. [22], found that the offspring of women who were randomly assigned to a high volume of moderate-vigorous intensity exercise in mid-late pregnancy were significantly lighter than infants born to women who did lower volumes of exercise. Despite vigorous intensity exercise having potential benefits in minimising maternal weight gain, trimester-specific evidence needs to be pooled for an improved synthesis of existing evidence before vigorous intensity exercise can safely be prescribed throughout pregnancy.

More specific guidelines are needed on vigorous intensity exercise in each trimester; and particularly in the final trimester, as this appears to be the most controversial within the literature. This is the first analysis of its kind to pool the evidence for studies reporting vigorous intensity exercise specifically in the third trimester. The primary aim of the study was to investigate the effects of vigorous intensity exercise during pregnancy on birth weight. The secondary aim was to investigate the effects on incidence of small for gestational age (SGA), low birth weight (LBW), prematurity, gestational age at delivery and maternal weight gain.

## Methods

This systematic review and meta-analysis was conducted according to the Preferred Reporting Items for Systematic Reviews and Meta-Analyses (PRISMA) guidelines [23]. Before the search was conducted, the review was registered with PROSPERO (International Prospective Register for Systematic Reviews), under registration number CRD42018102109 [24].

### Search strategy

Electronic searching of the PubMed, Medline, EMBASE, Cochrane Library, Web of Science and CINAHL databases was used to conduct the search up to November 2018. The Medical Subject Heading (MeSH) database, Boolean operators and truncation were employed to establish all related articles on exercise and pregnancy. The complete search strategy for each of the databases is available in Additional file 1. Only publications in English were included.

### Eligibility criteria

Studies included in the systematic review were randomised control trials (RCTs), quasi-experimental studies, cohort studies and case-control studies. The studies were required to include 1) an intervention or report of pregnant women (of any maternal age) performing vigorous exercise during gestation, 2) vigorous exercise reported in any trimester of pregnancy, 3) a comparator group of either lower intensity exercise or standard care, and 4) at least one of the following infant or maternal outcomes: birth weight, SGA, LBW, gestational age at delivery, preterm birth, or gestational weight gain. Studies reporting any type of vigorous physical activity were included in the review, including but not limited to: running, swimming, circuit training, interval training, weight lifting, or plyometrics. Studies were excluded if the sample was a population of women specifically with gestational diabetes mellitus, as infants born to women with this condition are more likely to have macrosomia [25]. However, studies reporting gestational diabetes as an outcome were included in the study.

### Definitions

The methodology of all articles on the effects of exercise in pregnancy on birth weight was reviewed in detail, to assess whether they met the definition of vigorous intensity exercise (regardless of the wording of the exercise intensity reported by the authors in the study). As ‘high-intensity’ exercise is considered a greater intensity than ‘vigorous’, both vigorous and high-intensity classifications are included in this review. Studies were included if they met any of the objective, subjective, or descriptive measures of vigorous or high-intensity exercise according to Exercise and Sports Science Australia’s position statement on exercise intensity terminology (Additional file 2) [15].

The reported incidence for SGA, preterm birth, gestational weight gain, birth weight and gestational age at delivery was based on the diagnosis provided by each study. However, in reference to the terms used throughout this review, the following standard definitions are used: 1) SGA is defined as birth weight below the 10th percentile of a population-specific birth weight versus gestational age plot [26]; 2) LBW is defined as birth weight less than 2500 g regardless of gestational age [26]; 3) Preterm birth is defined as a live birth < 37 completed weeks of gestation [27]; 4) Birth weight is defined as the first weight obtained after birth [27]; 5) Gestational age at delivery is defined as the number of completed weeks of gestation at time of delivery [27]; 6) Gestational weight gain is defined as the weight gained from a measure at a pre-conceptional visit to the last measured available weight during pregnancy abstracted from clinical records [28].

### Assessment of risk of bias

The Cochrane Risk of Bias for Randomized Controlled Trials was used to assess the risk of bias in the RCTs (Table 3) and the Newcastle-Ottawa Scale was used to assess the quality of cohort studies (Table 4) and case-control studies (Table 5) [45, 46]. For the Cochrane Risk of Bias for Randomized Controlled Trials, bias in each study is assessed as low, high or unclear risk across the domains of selection bias (random sequence generation and allocation concealment), reporting bias, other bias, performance bias, detection bias, and attrition bias. From these scores an overall quality assessment of low, unclear, or high-risk was provided. For the Newcastle-Ottawa Scale, quality is assessed from eight questions (one question which includes two parts) based on selection, comparability, as well as outcome for the cohort studies, and exposure for the case-control studies. From these scores an overall quality assessment is determined by the total of the scores out of nine. Two reviewers (CG and KB) conducted the evaluation separately. When there was a discrepancy, a third reviewer provided an evaluation (MN).

### Data collection process

Results of the searches were exported to EndNote X9 for removal of duplicates. Titles and abstracts were screened by CG, with any uncertainties verified by KB. The full-text of included studies were retrieved for data extraction and were reviewed in full by CG and KB. Data from the included studies were screened separately by two reviewers (CG and KB). The following information was extracted: study setting; population and participant demographics and baseline characteristics; intervention and control condition details; methodology; recruitment and study completion rates; outcomes and times of measurement; and information for assessment of the risk of bias.

### Statistical analysis

The primary outcome was the impact of vigorous intensity exercise on infant birth weight. Secondary outcomes were the impact of vigorous intensity on SGA, LBW, prematurity, gestational age at delivery and maternal weight gain. As recommended by Ioannidis, et al. [47], meta-analyses were conducted for all instances where two or more studies presented data on comparable participants, interventions, comparators and outcomes. We planned to assess the influence of vigorous exercise in each trimester, but sufficient data were only available for the third trimester. For example, we intended to assess birth weight when vigorous intensity exercise was stopped after the second trimester, but only one study reported vigorous intensity exercise stopping at the second trimester with birth weight as an outcome [40].

The metafor package [48] in R [49] was used to conduct random-effects multi-level meta-analyses. Multi-level meta-analyses produce less biased parameter estimates than averaging multiple outcomes within studies or arbitrarily selecting one outcome from a study [50].

Meta-analyses were conducted separately for each outcome. For continuous outcomes, unstandardised mean differences were calculated (e.g., birth weight in grams) to preserve the clinical significance of outcomes. For dichotomous outcomes (e.g., prematurity), a risk ratio was calculated. Pooled effect sizes were calculated using cluster-robust standard errors that corrected for correlations between effect sizes within studies [51].

When studies did not report means or standard deviations, we used the best available approximation from a systematic review of managing missing data in meta-analyses [52]. These approximations have been shown to reduce biases introduced from alternative approaches (e.g., list-wise deletion of studies). Where possible, planned moderation analyses were conducted for different study designs (prospective, retrospective, RCT) and different comparison conditions (vigorous vs. moderate exercise; vigorous vs. light exercise or less). In moderation analyses, standardised mean difference (Hedges’ g) were used for parsimony so multiple outcomes could be presented on the same forest plot. Finally, heterogeneity was assessed using a confidence-interval for *I*^2^ because point estimates of heterogeneity can be biased in small meta-analyses [53].

## Results

### Study selection

**Fig. 1:**
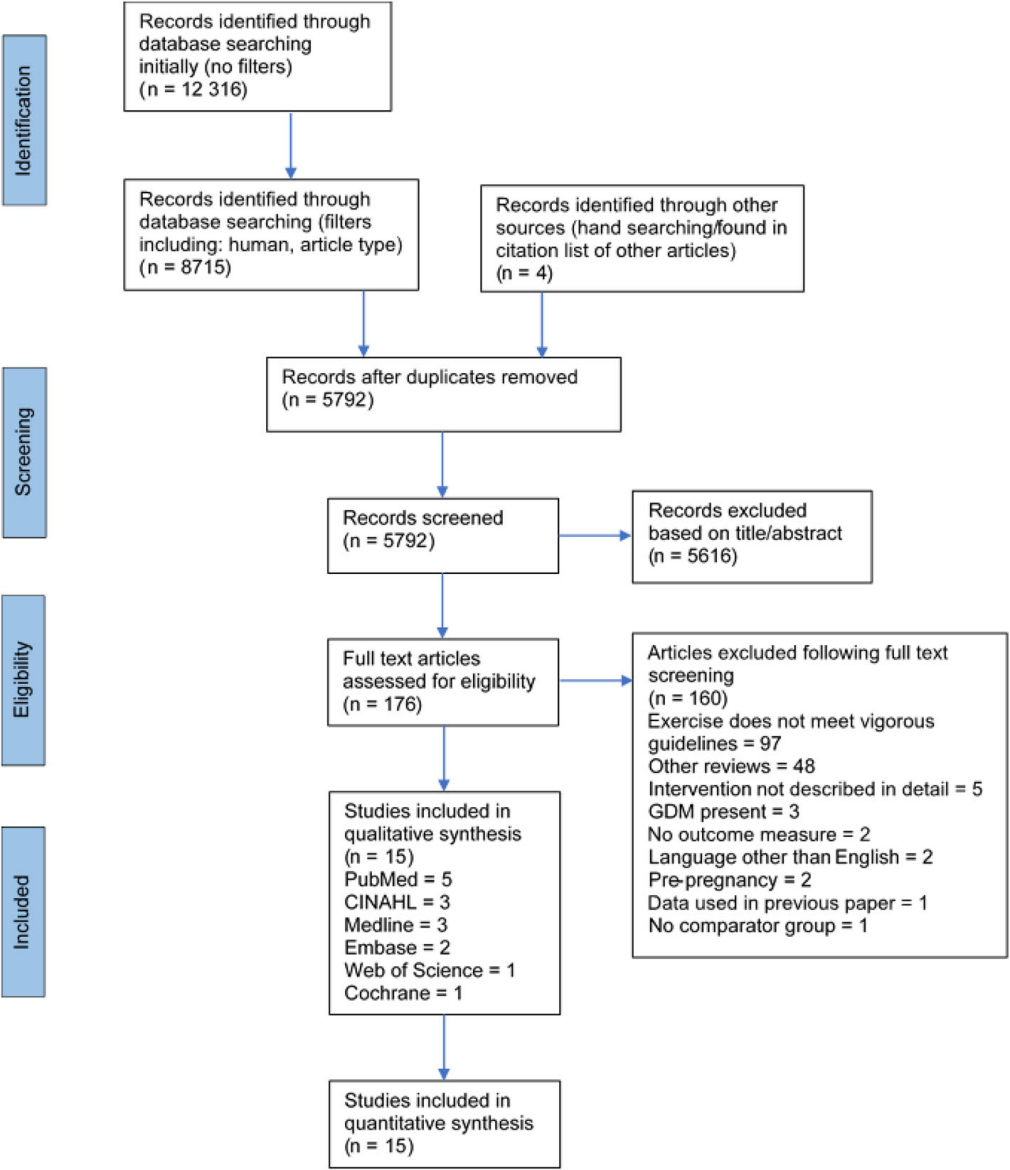
PRISMA flow diagram. GDM = gestational diabetes mellitus

In total, 12,316 articles were initially screened for inclusion in the study (Fig. 1). After filters were applied and duplicates were removed, 5792 articles were screened by title and abstract. The full texts of 176 articles were reviewed for eligibility criteria, and 15 studies met the criteria for inclusion in the systematic review. As such, five RCTs (*n* = 623) (Table 1) and ten cohort studies (*n* = 32,080) (Table 2) were included in the systematic review. Rose, et al. [40] and McCowan, et al. [42] were considered in the systematic review as we initially planned to compare trimesters however, these were the only studies which either stopped vigorous intensity exercise after the second trimester, or did not report vigorous intensity exercise in the third trimester. For this reason, they were not included in the meta-analysis. This removal left a total of eight cohort studies (*n* = 7225) and five RCTs (*n* = 623) in the statistical analysis.

**Table 1:** Characteristics of randomized controlled trials included in the systematic review and meta-analysis (*n* = 5)

	Subjects (n)				Intervention					Control	
Author, year, country	Total	Int	Con	Randomization GA (weeks)	Mode	Trimester	Frequency (times/week)	Duration (min)	Intensity	Intensity	MA outcomes reported
Ruchat, et al., 2012, Canada^a^ [29]	118	26	Low intensity group, *n* = 23, historical standard care, *n* = 45	16–20	Partially supervised walking sessions	3rd	3–4 (1x supervised, 3–4 unsupervised)	25–40	70% HRR	30% HRR	BW, GA at delivery, GWG, SGA
Bisson, et al., 2015, Canada [31]	50	25	25	< 14	Supervised aerobic and resistance training	3rd	3	60	70% MHR	Standard care	BW, GA at delivery, GWG, SGA
Cavalcante, et al., 2009, Brazil [32]	71	34	37	16–20	Supervised indoor water aerobics	3rd	3	50	70% MHR	No exercise	BW, GWG, prematurity, SGA
Hopkins, et al., 2010, New Zealand [33]	84	47	37	19	Fortnightly supervised home-based cycle ergometer	3rd	Maximum 5	40	65% VO_2_max	Standard care	BW, GA at delivery, SGA
Wang, et al., 2017, China [34]	300	150	150	10	Supervised cycle ergometer	3rd	3	30	3–5 30 s intervals at 75–85% MHR	Standard care	BW, GA at delivery, GWG, prematurity, SGA

*MA* Meta-analysis, *HRR* Heart rate reserve, *MHR* Maximum heart rate, *BW* Birth weight, *GA* Gestational age, *GWG* Gestational weight gain; prematurity, *SGA* Small for gestational age

^a^Data also published in Ruchat, et al. [30]

**Table 2:** Characteristics of cohort studies included in the systematic review and meta-analysis (*n* = 10)

	Subjects (n)				Exercise					Control	
Author, year, country	Total	Int	Con	Recruitment GA (weeks)	Mode	Tri	Frequency (times/week)	Duration (min)	Intensity	Intensity	MA outcomes reported
Bell, et al., 1995, Australia [35]	99	58	41	< 20	Any	3rd	≥3	≥3	“working up a sweat, getting puffed and at least 50% MHR”	No vigorous exercise prior to or during pregnancy	BW, SGA
Collings, et al., 1983, United States [36]	20	12	8	2nd trimester	Supervised cycle ergometer	3rd	3	40	65–70% VO_2_max	No exercise	BW, GWG
Magann, et al., 2002, United States [37]	455	238	217	< 20	Any	3rd	≥3	≥3	60–80% MHR	No exercise	BW, SGA, GA at delivery, Prematurity, GWG
Hegaard, et al., 2010, Denmark [38]	4458	176	Light intensity, *n* = 2384, sedentary, *n* = 1998	16	Any	3rd	Unable to evaluate	≥180	Moderate to heavy: “running, swimming, tennis, and competitive sports.”	Light intensity: “light gardening, playing table tennis”; sedentary: “mostly sitting”	BW, SGA
Sternfeld, et al., 1995, United States [39]	388	33	Moderate intensity, *n* = 53, light exercise, *n* = 55, sedentary, *n* = 242	< 20	Aerobic	3rd	≥3	≥20	“Vigorous walking” (specific intensity not reported)	Moderate intensity: aerobic, without vigorous intensity; light exercise: at least once per week but less than other groups; sedentary: no aerobic exercise	BW
Rose, et al., 1991, United States [40]	21,342	1264	Light intensity, *n* = 2127, sedentary, *n* = 17,951	2nd	Any	2nd	Not specified	Not specified	‘vigorous activity’	‘light or moderate activity’	BW, SGA
Kuhrt, et al., 2018, United Kingdom [43]	787	206	581	Retrospective	Running	3rd	≥1	n/a	n/a	No running	GA at delivery, prematurity
Zeanah, et al., 1993, United States [44]	173	18	69	Retrospective	Any	3rd	≥2	n/a	≥150 bpm	Moderate intensity: 130–149 bpm; Light intensity: ≤129 bpm	GWG, BW
Hall, et al., 1987, United States [41]	845	452	393	Not reported	Supervised machine-based resistance training, and cycling	3rd	3	45	85% MHR	No exercise	BW, GA at delivery
McCowan, et al., 2010, International [42]	3513	41	3472	15 weeks	Any	2nd	Daily	Not specified	“Exercise leading to heaving breathing or being out of breath”	Not specified	SGA

*Int* Intervention group, *Con* Control group, *Tri* Trimester, *MA* Meta-analysis, *HRR* Heart rate reserve, *GWG* Gestational weight gain, *BW* Birth weight, *MHR* Maximum heart rate; cardiorespiratory fitness, *MVPA* Moderate to vigorous physical activity, *IR* Insulin resistance; prematurity, *SGA* Small for gestational age, *GA* Gestational age

### Risk of bias

The risk of bias of the five RCTs are detailed in Table 3, and the quality assessment of the cohort and case-control studies are detailed in Tables 4 and 5, respectively. The overall risk of bias of the RCTs were mixed. All studies were considered low risk for reporting bias, performance bias blinding and detection blinding. However, attrition bias was poorly reported. The cohort studies scored higher in the quality assessment than the case-control studies. As expected in observational studies, there were mixed scores for the representativeness of the cohort, with instances of convenience sampling. However, all but one study used controls from the same representative cohort as the exposure group. The number of studies controlling for confounding factors was mixed, with four studies including no confounding variables in their statistical analyses (two cohort studies and two case-control studies).

**Table 3:** Cochrane Risk of Bias for Randomized Control Trials

	Selection bias Random sequence generation	Selection bias Allocation concealment	Reporting bias Selective reporting	Other bias Other sources of bias	Performance bias Blinding (participants & personnel)	Detection bias Blinding (outcome assessment)	Attrition bias Incomplete outcome data
Ruchat, et al. (2012) [29, 30]	L	U	L	L	H	L	H
Bisson, et al., (2015) [31]	L	L	L	L	L	L	L
Cavalcante, et al. (2009) [32]	L	L	L	L	L	L	L
Hopkins, et al. (2010) [33]	U	U	L	L	L	L	L
Wang, et al. (2017) [34]	L	L	L	L	L	L	L

**Table 4:** Newcastle-Ottawa Scale for cohort studies

	Selection				Comparability		Outcome			
	Representativeness of the exposed cohort	Selection of the non-exposed cohort	Ascertainment of exposure	Demonstration that outcome of interest was not present at start	Study controls for relevant primary confounder	Study controls for other secondary confounders	Assessment of outcome	Was follow up long enough for outcomes to occur	Adequacy of follow up of cohorts	Total
Bell, et al. (1995) [35]	0	0	0	1	1	1	0	1	0	4
Collings, et al., (1983) [36]	0	1	1	1	0	0	1	1	1	6
Magann, et al. (2002) [37]	0	1	0	1	1	1	1	1	1	7
Hegaard, et al. (2010) [38]	1	1	0	1	1	1	1	1	0	7
Sternfeld, et al. (1995) [39]	1	1	1	1	1	1	1	1	1	9
Rose, et al. (1991) [40]	0	1	0	1	1	1	1	1	1	7
Hall, et al., (1987) [41]	1	1	1	1	0	0	1	1	1	7
McCowan, et al. (2010) [42]	1	1	1	1	1	1	1	1	1	9

**Table 5:** Newcastle-Ottawa Scale for case-control studies

	Selection				Comparability		Exposure			
	Is the case definition adequate?	Representativeness of the cases	Selection of controls	Definition of controls	Study controls for relevant primary confounder	Study controls for other secondary confounders	Assessment of exposure	Same method of ascertainment for cases and controls	Non-response rate	Total
Kuhrt, et al. (2018) [43]	0	0	1	1	0	0	0	1	0	3
Zeanah, et al., (1993) [44]	0	0	1	0	0	0	0	1	0	2

### Intensity

The use of exercise intensity terminology was varied. Three studies reported moderate intensity exercise, but the description indicated it was vigorous intensity exercise. A study by Bell, et al. [35] reported women were achieving at least 50% MHR, which would not traditionally be classified as vigorous exercise. However, they also reported the women were required to achieve an intensity that elicited ‘getting puffed’, which according to the intensity definition by Norton, et al. [15] has a relative intensity of between 70 and 90% MHR. Furthermore, the studies by Ruchat et al. [29, 30] and Cavalcante Sergio et al. [32] reported a moderate intensity group of 70% heart rate reserve and MHR respectively, which is classified as vigorous intensity exercise. As such, it was deemed that all three studies met the criteria for vigorous intensity and were included in the systematic review and meta-analysis.

### Birth weight, low birth weight and small for gestational age

**Fig. 2:**
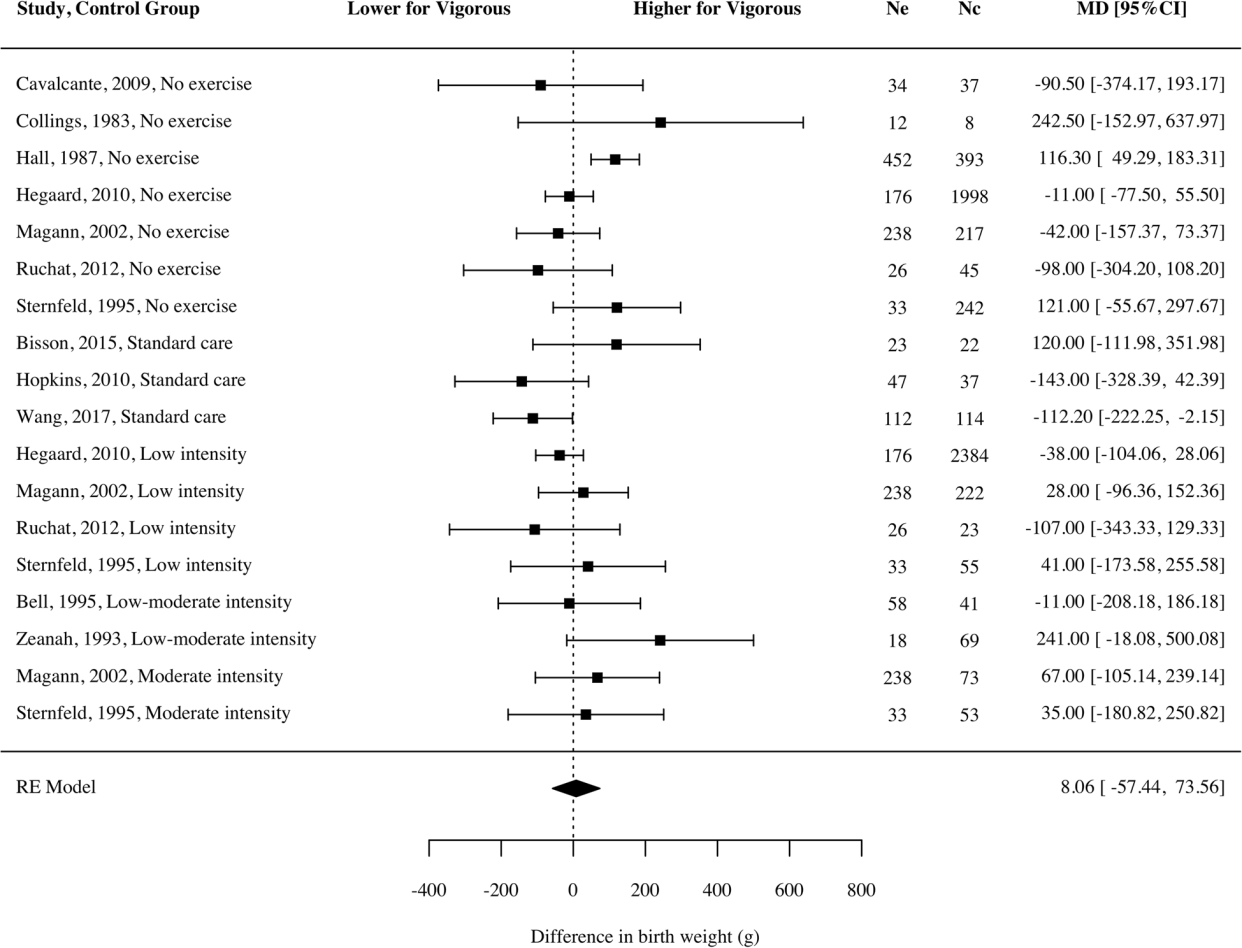
Mean difference of birth weight for women participating in vigorous intensity exercise compared to a control

**Fig. 3:**
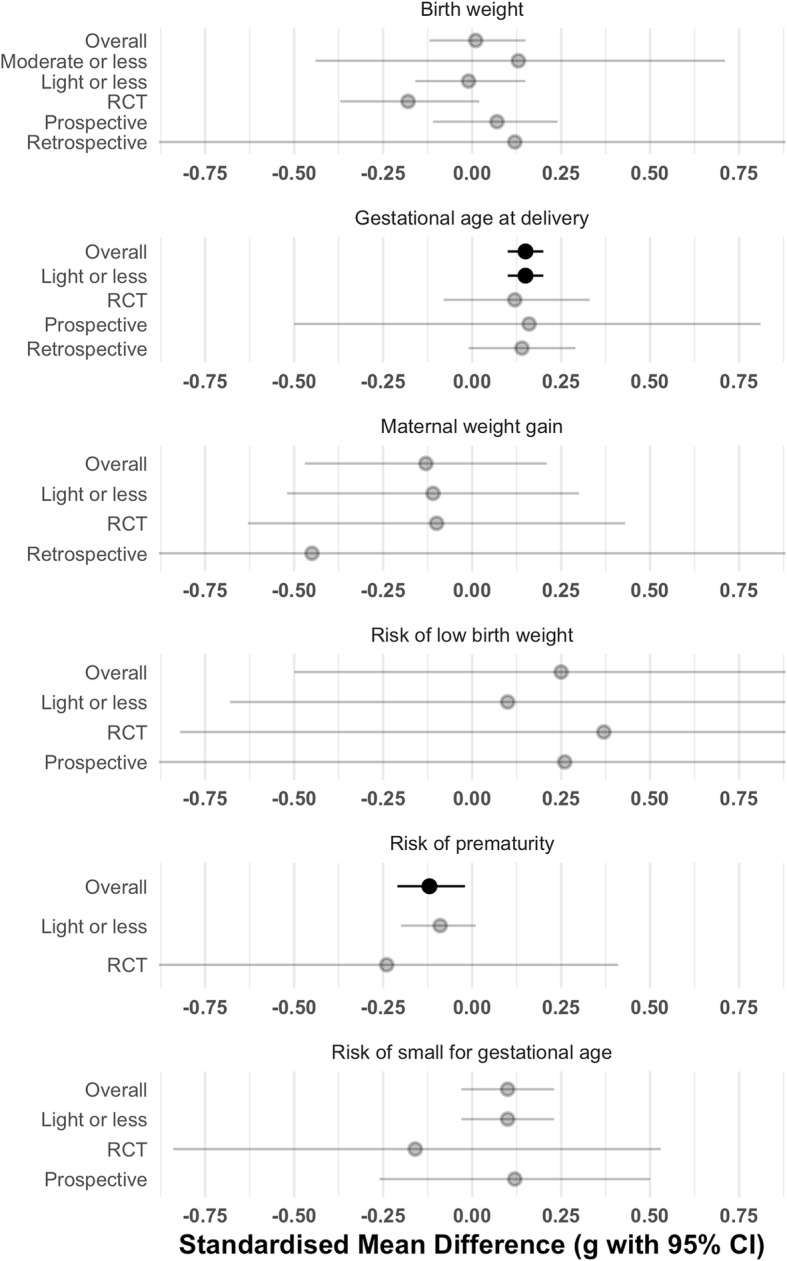
Moderation analysis of each of the variables according to study design and comparison condition. Significant values are highlighted in black, and non-significant values are highlighted in grey

No significant difference occurred in birth weight for babies of mothers who engaged in vigorous physical activity and those who did not (Fig. 2; mean difference 8.06 g, 95% CI − 57.44 to 73.55, *p* = 0.79, *g* = 0.01, *n* = 8006, k = 12, *I*^*2*^ = 53.92 [0, 85.03]). Fig. 3 shows this finding was consistent across moderation analyses with no significant pooled mean differences for any designs (i.e., retrospective, prospective, RCT) or comparison conditions (i.e., women who did moderate intensity exercise or less, and those who did light exercise or less) (Additional file 3).

**Fig. 4:**
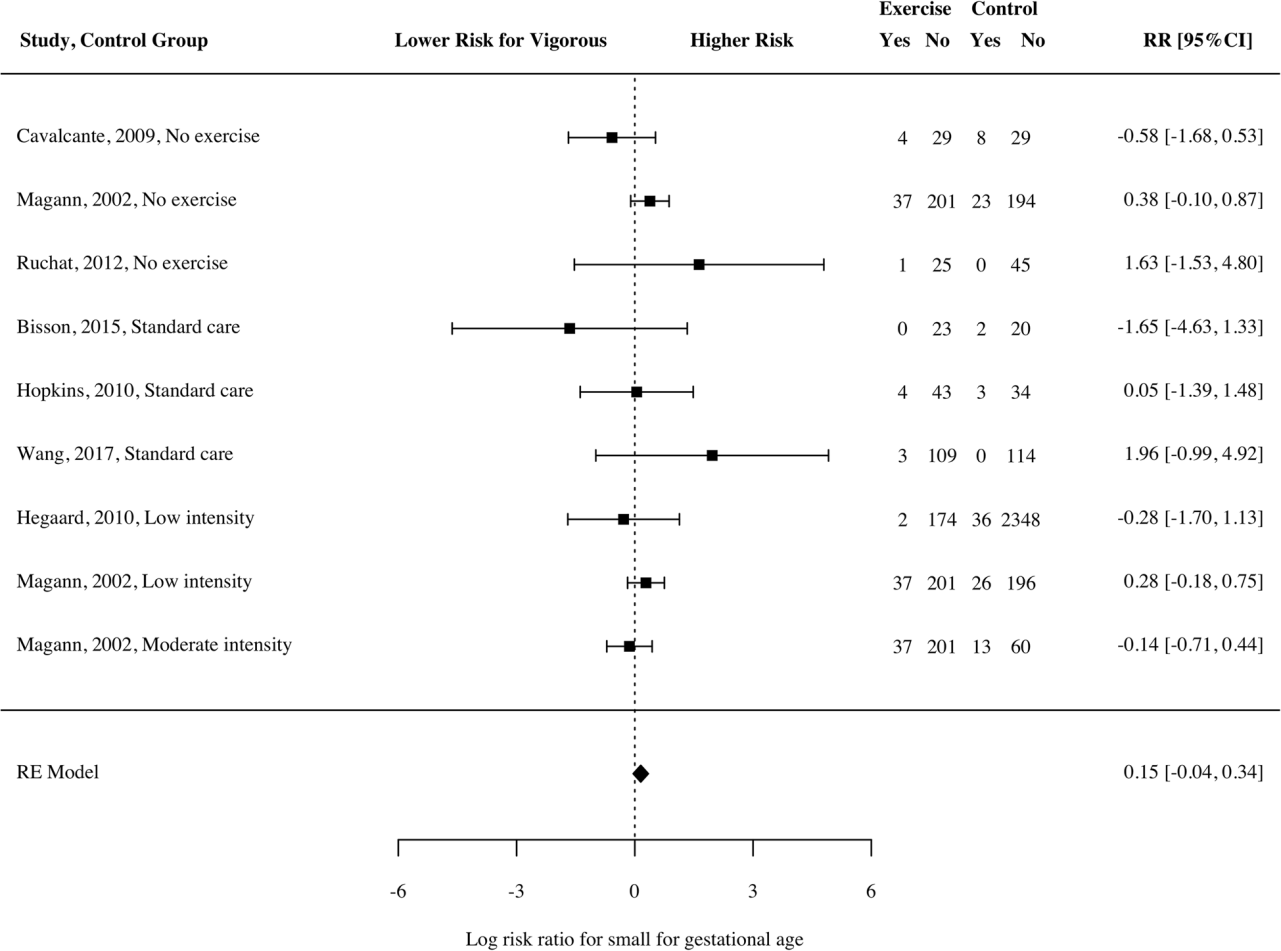
Log risk ratio of small for gestational age infant for women participating in vigorous intensity exercise compared to a control

**Fig. 5:**
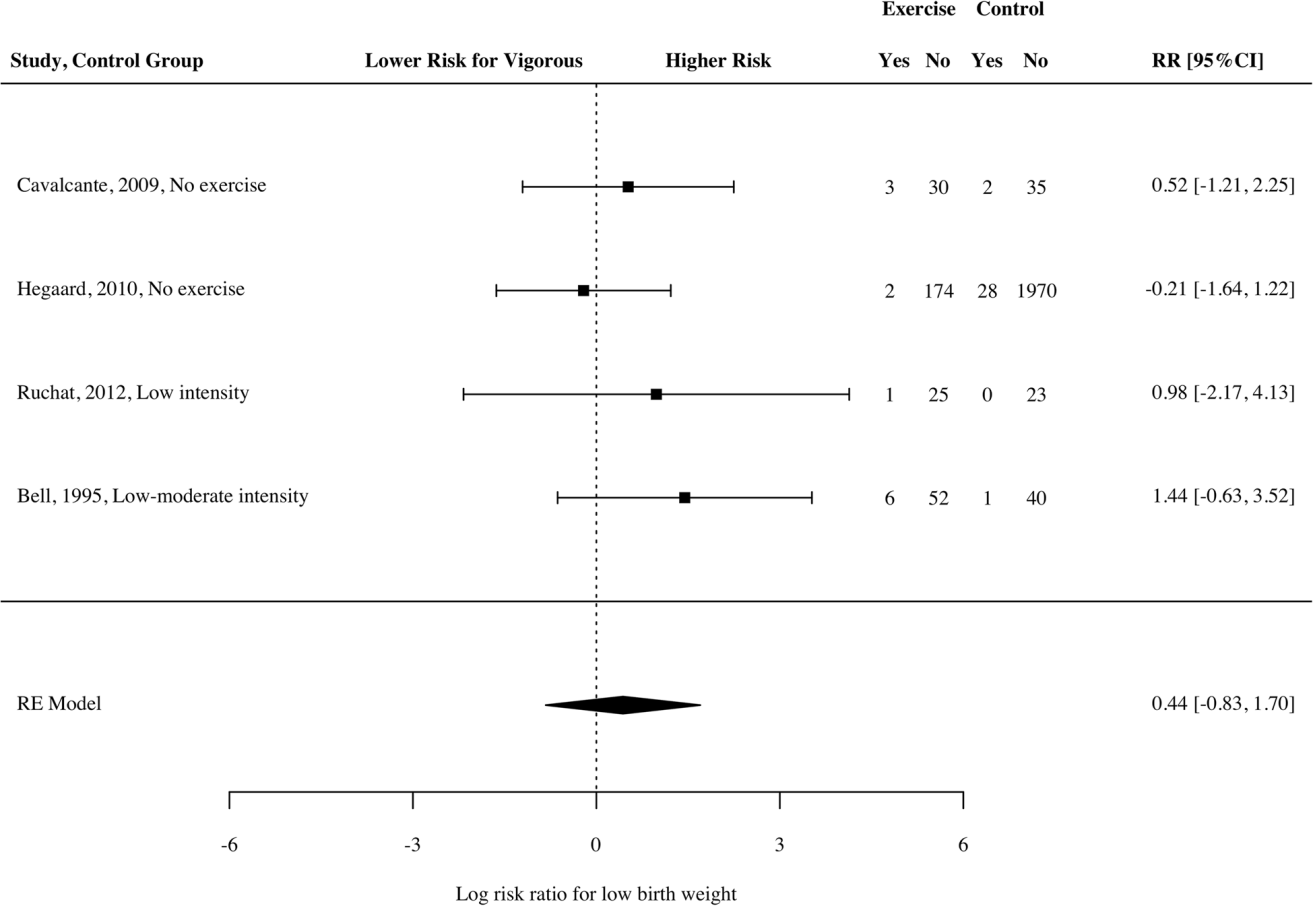
Log risk ratio of low birth weight infant for women participating in vigorous intensity exercise compared to a control

Five studies [31–34, 37] used a definition of birth weight relative to gestational age (i.e., SGA), and four studies used < 2500 g as the definition of LBW [9, 29, 32, 38]. There was no significant increase in risk of SGA in those who undertook vigorous exercise compared to those who did not (Fig. 4; *RR* = 0.15, 95% CI − 0.06 to 0.35, *p* = 0.13*, n* = 4504, k = 7, *I*^*2*^ = 1.11 [0, 90.75]). This finding was consistent when looking only at studies that used a comparison condition of light exercise or less, and when moderating for study design (separating RCTs and prospective studies). Similarly, there was no significant increase in risk of LBW (Fig. 5; *RR* = 0.44, 95% CI − 0.83 to 1.7, *p* = 0.35*, n* = 2454, k = 4, *I*^*2*^ = 0 [0, 91.81]). This was also consistent using light exercise as the comparator, and when exploring RCTs and prospective studies separately. However, a three-fold risk of delivering a SGA infant was observed in a prospective study of 3513 primiparous mothers from Australia, New Zealand, United Kingdom and Ireland who reported daily vigorous intensity exercise in the first 15 weeks of pregnancy [42]. This study was not included in the meta-analysis as it did not monitor vigorous intensity exercise throughout pregnancy.

### Gestational age at delivery and prematurity

**Fig. 6:**
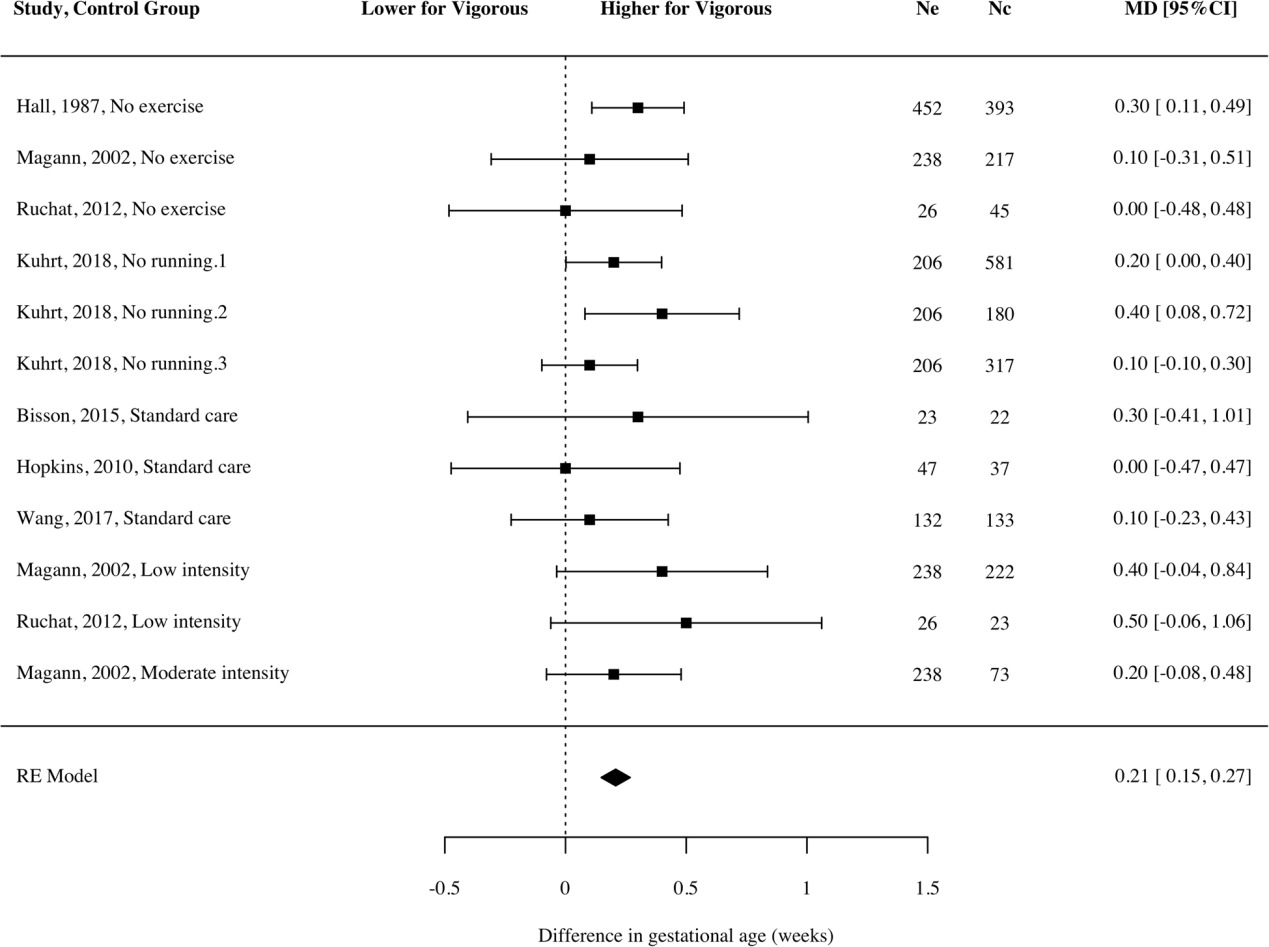
Mean difference of gestational age at delivery for women participating in vigorous intensity exercise compared to a control

A small but significant increase was observed in gestational age at delivery of babies of women who engaged in vigorous intensity exercise (Fig. 6; mean difference = 0.21 weeks; 95% CI 0.15 to 0.27, *g* = 0.15, *p* < 0.001, *n* = 4281, k = 7, *I*^*2*^ = 0 [0, 68.52]). Those who participated in vigorous intensity exercise gave birth at an average of 39 + 4 weeks vs. 39 + 3 weeks in the control groups. In all studies, women who performed vigorous exercise were compared with those who did light or no exercise (i.e., there was no moderate intensity comparison group). Effect sizes were similar, but findings were not significant when exploring RCTs (mean difference = 0.16 weeks; *n* = 443, k = 4), prospective (mean difference = 0.26 weeks; *n* = 2071, k = 2) and retrospective (mean difference = 0.18 weeks; *n* = 1767, k = 2) studies separately, potentially due to the smaller number of studies and participants in each meta-analysis.

**Fig. 7:**
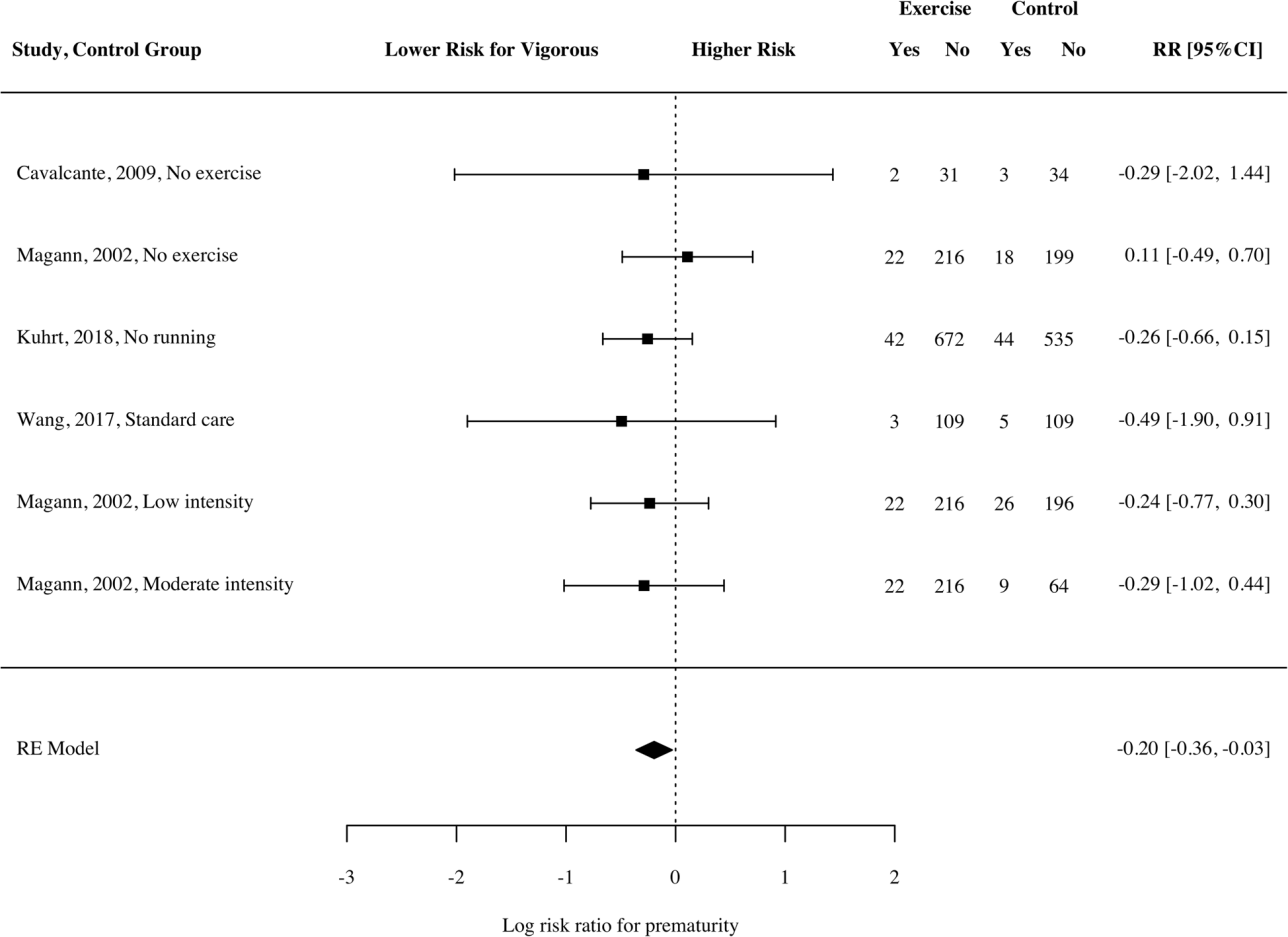
Log risk ratio of prematurity for women participating in vigorous intensity exercise compared to a control

Similarly, a small, but significant, reduced risk of prematurity existed in babies of mothers who engaged in vigorous physical activity (Fig. 7; *RR* = − 0.20; 95% CI − 0.36 to − 0.03, *p* = 0.03, *n* = 3025, k = 4, *I*^*2*^ = 0 [0, 86.02]). These findings did not replicate when examining only the two RCTs (*RR* = − 0.41; 95% CI − 1.64 to 0.82, *p* = 0.15, *n* = 312) or when using only light intensity exercise as a comparison (*RR* = − 0.16; 95% CI − 0.32 to 0.01, *p* = 0.05, *n* = 1644, k = 3).

### Maternal weight gain

**Fig. 8:**
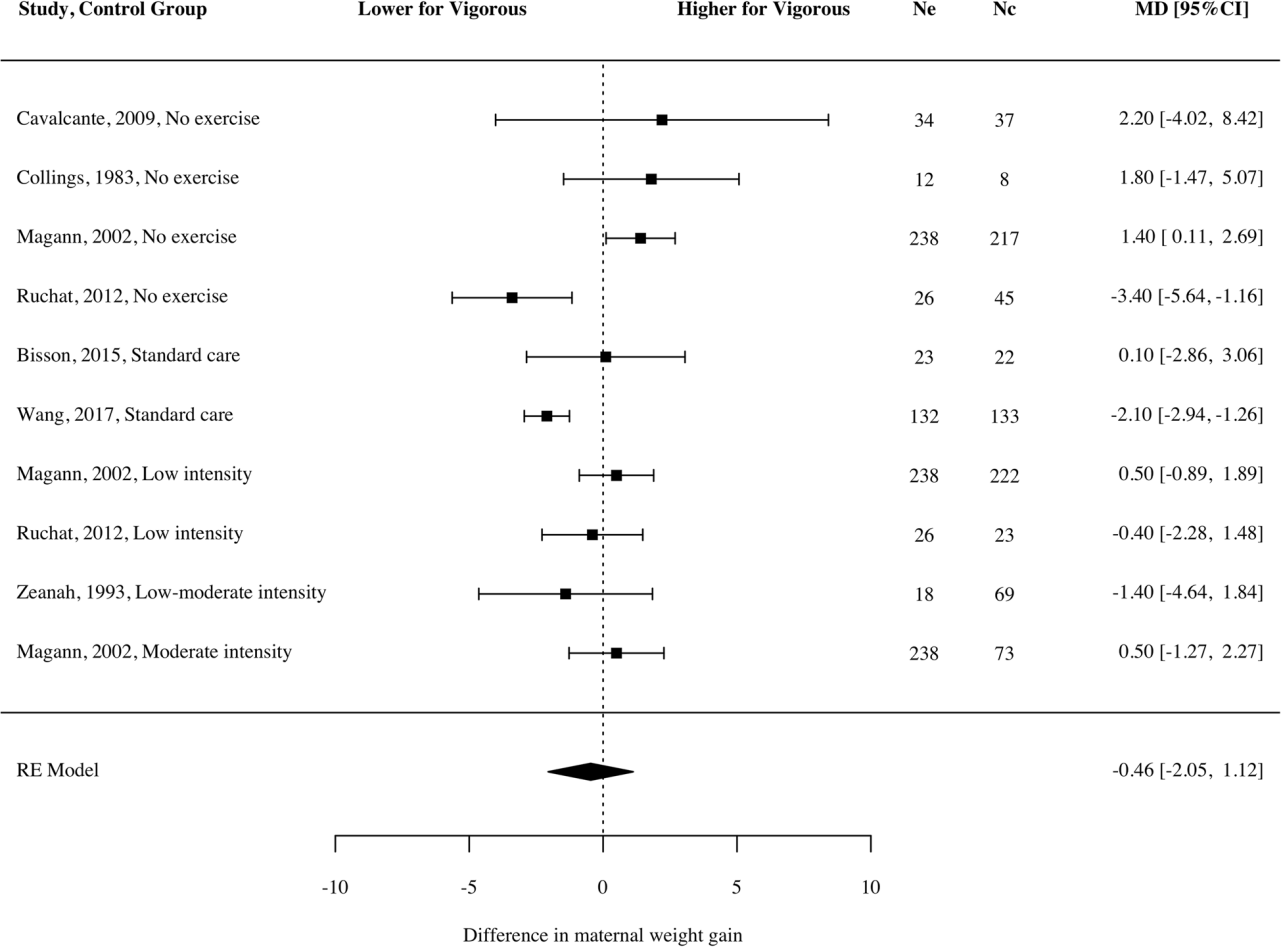
Mean difference of maternal weight gain for women participating in vigorous intensity exercise compared to a control

No significant difference in maternal weight gain was apparent for women who engaged in vigorous intensity exercise (Fig. 8; mean difference = − 0.46 kg, 95% CI − 2.05 to 1.12, *g* = − 0.13, *p* = 0.5, *n* = 1834, k = 7, *I*^*2*^ = 68.94 [0, 95.2]). These findings were consistent across study design and comparison condition (see Fig. 3).

The RCTs targeting overweight and obese pregnant women did show a significant reduction in maternal weight gain compared to a control group [31, 34]. Further, one of these studies reported an increase in fat percentage in the control group compared with the exercise group [31]. The study by Ruchat et al. [29, 30] reported 53% of women in a non-exercising control group had excessive weight gain (average weekly weight gain > 0.5 kg), compared with only 31% in the vigorous intensity group, and 35% in the light intensity group.

### Adverse events

All studies were conducted in low-risk women, with exclusion criteria which included conditions such as cervical insufficiency, presence of chronic disease, or any contraindication to exercise. The five RCT’s included in this review suggest no increased risk of an adverse event occurring as a result of vigorous exercise training. The study by Wang, et al. [34] indicated that 38 participants dropped out of the exercise group, and 36 from the standard care group. The main reason was attributed to an unwillingness to participate further. However, four women in the vigorous intensity exercise group had miscarriages, and there were three miscarriages and one fetal death in utero for women in the standard care group. As such, miscarriage and fetal death in utero was not different between the exercise and control group. There was only one miscarriage reported in the study by Bisson, et al. [31], and this occurred in the standard care group. Three women in the standard care group in the study by Hopkins, et al. [33] met exclusion criteria in late pregnancy, as they developed pre-eclampsia and preterm labor (< 30 weeks gestation). No adverse events occurred in the women who dropped out of the study by Cavalcante Sergio, et al. [32] or Ruchat et al. [29, 30]. It is important to note that the interventions in all RCTs were often not commenced until either the latter stage of trimester one, or the start of trimester two. As such, it is not possible to determine the effects of vigorous intensity exercise on adverse events such as miscarriage, when the first trimester is the most vulnerable time for these events [54].

## Discussion

The findings from the meta-analysis indicated no significant difference in birth weight from mothers who completed vigorous intensity exercise in the third trimester compared with controls. Further, no significant mean difference was observed between vigorous intensity exercise and control groups on incidence of SGA, LBW, or maternal weight gain. However, women undertaking vigorous intensity to third trimester did have a small, but significant, increase in gestational age at delivery and decreased risk of prematurity.

Clapp, et al. [55] suggested that the intermittent periods of hypoxia inherent in vigorous exercise, as blood is re-directed to the working muscles, may actually be advantageous to the fetus in the first and second trimester, as this is the time when the growth of the placenta at the level of the intermediate villi is greatest [55]. Indeed, periods of hypoxia can increase placenta vascularisation through angiogenesis in the placenta [56]. As such, vigorous intensity exercise in the first and second trimesters can result in a healthier placenta. However, the needs of the fetus are greater in the third trimester, with blood flow to the uterus increasing from 50 mL/min in the first trimester to 500 mL/min in the third trimester [57]. It is postulated that fetus compensatory sympathetic responses are in place to deal with a reduction in blood flow [58], exemplified by what occurs transiently during vigorous exercise. This is supported in the study by Collings, et al. [36], who reported an increase in fetal heart rate responses during and after vigorous intensity exercise. Indeed, the findings from this meta-analysis indicate an absence of detrimental effects of vigorous intensity exercise in the third trimester on markers which may reflect outcomes of reduced blood flow, such as incidence of SGA, LBW and prematurity. There also appeared to be no difference between groups in the women who suffered miscarriages in the RCTs. However, there is a lack of detail in the reporting of adverse events in the cohort and case-control studies, and caution is required.

A meta-analysis by Leet and Flick [59] found endurance exercisers who continued exercise into the third trimester delivered infants who weighed 212.2 g less than active controls (in six studies), and 436.5 g less than sedentary controls (in two studies). However, the weight loss was insufficient to be considered as a diagnosis of SGA. Notably, only one of these studies provided exercise descriptions that were considered adequate to be defined as vigorous intensity exercise. Reduced birth weight without diagnosis of SGA was also found in two other reviews [60, 61]. The lower birth weight that is shown in some studies is thought to be due to reduced fetal fat deposition, rather than a reduction in lean mass [22]. However, it would seem pertinent to suggest that women who are carrying fetuses on the lower end of the weight chart in the later stages of pregnancy should be cautious about undertaking vigorous exercise in the third trimester, as, although not significant, the pooled results from the RCTs demonstrated slightly lower birth weight than controls.

Every paper reported vigorous intensity exercise in a different way, making it difficult to compare studies. It is hard to differentiate if it is intensity, frequency, duration, or volume (total exercise workload which can be a factor of intensity, frequency and duration) of exercise sessions, that contributes to the lower birth weight reported in some of the studies. Bell, et al. [35] identified that frequency of vigorous intensity exercise may relate to birth weight, with findings indicating a decrease in birth weight with increasing number of exercise sessions (3 sessions = 3682 g birth weight, and 5/6/7 sessions = 3049 g birth weight). On the other hand, the study by Kuhrt, et al. [43] showed that neither average weekly kilometers (i.e., volume), or trimester that women ran to, influenced birth weight percentiles. The retrospective survey by Zeanah and Schlosser [44] also showed no effect of higher volume (> 80 min/week) or higher intensity (> 150 bpm) exercise during third trimester, on birth weight. The study by Takami, et al. [62], divided 92,796 women into very low, low, medium and high levels of physical activity, based on met-hours per week. The equation for met-hours per week uses a weighting for intensities, therefore looking at volumes of exercise rather than specific intensities. However, this study found no detrimental effects of high-volume exercise on infant outcomes. Conversely, a significant increase was reported in prematurity in the very low volume exercise group. This is in line with our own findings, which found a reduction in prematurity in women undertaking vigorous intensity exercise. Rather than vigorous intensity exercise having a physiological effect on reducing prematurity, it is more likely to indicate women participating in vigorous intensity exercise and/or choosing to be involved in an exercise research study, are likely to be healthier with lower risk pregnancies.

It is important to note that most studies in this systematic review reported intensities below 90% MHR (or equivalent). Indeed, according to the terminology reported in Norton, et al. [15], most studies included in this review described exercise considered vigorous intensity (< 90% MHR) and *not* high-intensity (≥90% MHR). This is significant to highlight as a study in elite athletes showed normal fetal heart rate responses to an acute bout of exercise conducted at 23–29 weeks of gestation, *until* the intensity reached above 90% MHR [63]. Only two of the seven athletes reached an intensity greater than 90% MHR, and in both of these athletes the mean uterine artery blood flow was less than 50% of the initial value with fetal bradycardia occurring (indicating fetal distress). However, fetal heart rate returned to normal upon cessation of the exercise. It is not clear what the long-term impact of this transient fetal bradycardia from acute strenuous exercise is. It could be that this extreme high-intensity exercise undertaken by some women is what is driving the tendency to lower birth weight in some studies. The study by Kardel and Kase [64], did report women reaching heart rates of 170–180 bpm (likely equivalent of greater than estimated 90% MHR) in two exercising groups (one with higher volume). This study reported measuring fetal heart rate and movement after a 10-min interval training session, assessed 6–7 times throughout the pregnancy, and used as a prognostic value for detection of fetal distress and antenatal hypoxia. However, the results from these measures are not reported in the article. As both studies were conducted with a small sample size, the impact of exercising at levels above 90% MHR warrants further investigation. Three studies included in this review demonstrated no negative effects of vigorous intensity exercise on fetal heart response [36, 41] and mean uterine arteries pulsatility index [31].

Due to the difficulty in accurate assessment of MHR during pregnancy (as a result of haemodynamic changes), associations of exercise intensity with ratings of perceived exertion are recommended [65]. The use of non-pregnant intensity guidelines [15] as an inclusion criteria for vigorous intensity exercise studies in this review is likely to represent pregnant women completing exercise at an intensity higher than vigorous intensity guidelines in non-pregnant guidelines. However, the purpose of this meta-analysis is to demonstrate the safety of an intensity that is likely prescribed as vigorous intensity exercise in research and clinical practice. As such, the lack of adverse events using non-pregnant vigorous intensity guidelines (i.e. the upper limit) provides reassurance of the safety of this intensity of exercise. Future research should validate pregnancy specific target heart rates throughout each trimester of pregnancy, alongside the varying changes in maternal haemodynamics, so the safety of adjusted intensities can be assessed.

It has been recommended by the Canadian Guidelines Consensus Panel for Physical Activity Throughout Pregnancy that chronic high-intensity exercise, above the target heart rates recommended, is only undertaken in a monitored environment [18]. Of the 15 included studies, only five reported exclusively supervised exercise sessions. Whilst these studies are considered vigorous intensity, not high-intensity exercise, the lack of adverse events in the studies that reported unsupervised exercise sessions should provide reassurance as to the safety of this type of exercise in most low-risk pregnancies.

Moderate intensity exercise is well reported to reduce gestational weight gain in normal weight, overweight and obese pregnant women [66]. However, the lack of benefit of vigorous intensity exercise on maternal weight gain in this meta-analysis is an interesting finding. The lack of additional benefit of maternal weight gain may suggest that vigorous intensity exercise in the third trimester is not necessary above and beyond moderate intensity exercise. It is important to note that the two RCTs in this review that recruited a cohort of overweight and obese pregnant women, did in fact find a benefit of vigorous intensity exercise on maternal weight gain compared to a control group [31, 34]. This perhaps indicates a benefit of vigorous intensity exercise in limiting maternal weight gain in overweight and obese populations, rather than in healthy weight women. Future research should identify any additional benefits on infant and maternal outcomes of vigorous intensity exercise in the third trimester, such as to antenatal anxiety and depression and gestational diabetes. It is also pertinent that studies conducted during pregnancy should document and report all adverse events occurring throughout the pregnancy and birth. Indeed, the original design of this systematic review was to compare the effects of vigorous intensity exercise ceased at each trimester, and the subsequent benefit or detriment of continuing vigorous exercise into the third trimester. Unfortunately, a lack of evidence precluded this sub-analysis.

### Strengths and limitations

There are both strengths and limitations to this meta-analysis. The main strengths of the paper are that it is the first of its kind to pool the evidence for studies reporting vigorous intensity exercise specifically in the third trimester. Further, by including both randomized, cohort and case-control studies we have been able to capture the scope of evidence in this area. Indeed, by doing so we have been able to identify an important discrepancy in reporting of lower birth weight in RCTs compared with cohort and case-control studies. However, the heterogeneity of the research designs is also a limitation in synthesising the evidence [67]. A random effects meta-analysis attempts to account for this by estimating the effects from similar interventions that operate on a similar outcome. The results from the moderator analyses did not demonstrate significant heterogeneity, however it is acknowledged that samples were small in some of these analyses. While point estimates of heterogeneity were often modest, the small number of studies meant the confidence intervals for heterogeneity were very wide. This means there may be heterogeneity in the outcomes that could not be explained by the studies in this review.

Further research on the effects of vigorous intensity exercise on maternal and infant outcomes is still needed, particularly in separating the benefits or detriments of high-intensity exercise versus high volume of exercise in the third trimester of pregnancy. Thorough documentation of adverse events should be prioritised, and future studies should also examine placenta function and growth in combination with birth weight. More evidence is needed on the impact of higher intensity on birth outcomes in elite athletes, who are the population likely to be exercising at > 90% MHR [68]. It is also important to note that in the studies included in this systematic review, the mode of exercise was not always reported. However, in most cases the vigorous intensity mode was aerobic exercise. Future research is still needed to assess the safety of high-intensity resistance training regarding changes in musculature (such as pelvic floor dysfunction and diastasis recti) during pregnancy.

## Conclusions

The findings from this meta-analysis indicate that vigorous intensity exercise during the third trimester appears not to compromise birth outcomes for most low-risk pregnancies. Moreover, women undertaking vigorous intensity exercise had a significantly lower risk of prematurity. On the other hand, the meta-analysis did identify that RCTs showed a non-significant reduction in birth weight, which was not replicated in the cohort studies. However, this did not translate to a significantly increased risk of infants born small for gestational age. If the fetus is on the lower end of the birth weight chart, it may therefore be safer to suggest only moderate intensity exercise be undertaken in the third trimester. There was also no added benefit of vigorous intensity exercise over moderate intensity exercise or standard care on maternal weight gain in healthy weight women. However, vigorous intensity exercise did reduce maternal weight gain in overweight and obese pregnant women. Without a higher quality of evidence, any vigorous intensity exercise program during pregnancy should be individualised and conducted with guidance from an exercise professional and medical practitioner. Pregnant women should avoid exercising at a perceived exertion relative to ≥90% MHR, until further research can confirm its safety. The findings from this meta-analysis will help guide women and practitioners in prescribing vigorous intensity aerobic exercise throughout all trimesters of pregnancy.

## Additional files

Additional file 1 Systematic Review Search Terms (DOCX 15 kb)

Additional file 2 Exercise and Sports Science Australia position statement on physical activity and exercise intensity terminology. Reproduced with permission from Norton et al. [15]. (PNG 151 kb)

Additional file 3 Results from all overall and moderation meta-analyses. (DOCX 15 kb)

## Data Availability

All data generated or analysed during this study are included in this published article [and its supplementary information files].

## Abbreviations

ACSM: American College of Sports Medicine
LBW: Low birth weight
MeSH: Medical Subject Heading
MHR: Maximum heart rate
PRISMA: Preferred reporting items for systematic reviews and meta-analyses
PROSPERO: International Prospective Register for Systematic Reviews
RCT: Randomised control trials
SGA: Small for gestational age
