# Supplementary material for: The effects of vigorous intensity exercise in the third trimester of pregnancy: a systematic review and meta-analysis
Source: BMC Pregnancy Childbirth. 2019 Aug 7;19:281. doi: 10.1186/s12884-019-2441-1 (PMC6686535; doi:10.1186/s12884-019-2441-1)
Supplement: Supplementary file 1 — Systematic Review Search Terms (DOCX 15 kb) [file 12884_2019_2441_MOESM1_ESM.docx]

Additional file 1. Systematic Review Search Terms

**PubMed**

**Population**

Key Words:

pregnan* OR gestation* OR trimester* OR ''expect* mother'' OR ''expect* woman*'' OR ''expect* women*'' OR ''prenatal care''

MeSH:

"Pregnant Women"[Mesh] OR "Pregnancy"[Mesh] OR "Pregnancy Trimesters"[Mesh] OR "Pregnancy Trimester, Third"[Mesh] OR "Pregnancy Trimester, Second"[Mesh] OR "Pregnancy Trimester, First"[Mesh] OR "Prenatal Care"[Mesh]

**Intervention**

Key Words:

exercise* OR exercising OR "physical activity" OR "physical activities" OR "physical exert*" OR "physical fitness" OR "fitness train*" OR running OR sport* OR "physical train*" OR "exercis* therap*" OR "exercis* movement technique*" OR "physical conditioning"

MeSH:

"Exercise"[Mesh] OR "Exercise Movement Techniques"[Mesh] OR "Exercise Therapy"[Mesh] OR "High-Intensity Interval Training"[Mesh] OR "Physical Exertion"[Mesh] OR "Physical Fitness"[Mesh] OR "Running"[Mesh] OR "Sports"[Mesh] OR "Physical Conditioning, Human"[Mesh] OR "Plyometric Exercise"[Mesh] OR "Circuit-Based Exercise"[Mesh] OR "Resistance Training"[Mesh] OR "Leisure Activities"[Mesh]

**Outcome**

Key Words:

"infant* weigh*" OR "infant* size" OR "baby weigh*" OR "baby size" OR "babies weigh*" OR "babies size" OR "neonat* weigh*" OR "neonat* size" OR "neo-nat* weigh*" OR "neo-nat* size" OR "newborn* weigh*" OR "newborn* size*" OR "new-born* weigh*" OR "new-born* size" OR "new born* weigh*" OR "new born* size" OR "fetal* weigh*" OR "fetal* size" OR "foetal* weigh*" OR "foetal* size" OR "fetus* weigh*" OR "fetus* size" OR "birth* weigh*" OR "birth* size" OR birthweigh* OR "fetal* growth retardation" OR "foetal* growth retardation" OR "fetal* hypoxia" OR "foetal* hypoxia" OR "infant, low birth weigh*" OR prematur* OR "placenta* circulation"

MeSH:

"Infant, Extremely Low Birth Weight"[Mesh] OR "Infant, Very Low Birth Weight"[Mesh] OR "Infant, Low Birth Weight"[Mesh] OR "Birth Weight"[Mesh] OR "Fetal Weight"[Mesh] OR "Fetal Hypoxia"[Mesh] OR "Fetal Growth Retardation"[Mesh] OR "Infant, Extremely Premature"[Mesh] OR "Infant, Premature"[Mesh] OR "Placental Circulation"[Mesh] OR "Fetal Development"[Mesh]

**CINAHL**

**Population**

Key Words:

pregnan* OR gestation* OR trimester* OR ''expect* mother'' OR ''expect* woman*'' OR ''expect* women*'' OR ''prenatal care''

Headings:

(MH "Pregnancy Trimesters") OR (MH "Pregnancy") OR (MH "Pregnancy Trimester, Third") OR (MH "Pregnancy Trimester, Second") OR (MH "Pregnancy Trimester, First") OR (MH "Expectant Mothers") OR (MH "Prenatal Care")

**Intervention**

Key Words:

exercise* OR exercising OR "physical activity" OR "physical activities" OR "physical exert*" OR "physical fitness" OR "fitness train*" OR running OR sport* OR "physical train*" OR "exercis* therap*" OR "exercis* movement technique*" OR "physical conditioning"

Headings:

(MH "Exercise") OR (MH "Resistance Training") OR (MH "Therapeutic Exercise") OR (MH "Exercise Intensity") OR (MH "Plyometrics") OR (MH "Aerobic Exercises") OR (MH "Anaerobic Exercises") OR (MH "Leisure Activities") OR (MH "Physical Activity") OR (MH "Physical Fitness") OR (MH "Sports")

**Outcome**

Key Words:

((infant* OR baby OR babies OR neonat* OR neo-nat* OR newborn* OR new-born* OR "new born*" OR fetal* OR foetal* OR fetus* OR foetus* OR birth*) N3 (weigh* OR size OR "growth retardation" OR premature OR "low birth weigh*" OR hypoxi*)) OR birthweight OR prematur* OR "placenta* circulation"

Headings:

(MH "Birth Weight") OR (MH "Infant, Very Low Birth Weight") OR (MH "Infant, Low Birth Weight") OR (MH "Fetal Weight") OR (MH "Fetal Circulation") OR (MH "Fetal Growth Retardation") OR (MH "Infant, Premature") OR (MH “Fetal Development”) OR (MH “Parity”) OR (MH “Infant, Small for Gestational Age”)

**Medline**

**Population**

Key Words:

pregnan* OR gestation* OR trimester* OR ''expect* mother'' OR ''expect* woman*'' OR ''expect* women*'' OR ''prenatal care''

MeSH:

(MH "Pregnant Women") OR (MH "Pregnancy") OR (MH "Pregnancy Trimesters") OR (MH "Pregnancy Trimester, First") OR (MH "Pregnancy Trimester, Second") OR (MH "Pregnancy Trimester, Third") OR (MH "Prenatal Care")

**Intervention**

Key Words:

exercise* OR exercising OR "physical activity" OR "physical activities" OR "physical exert*" OR "physical fitness" OR "fitness train*" OR running OR sport* OR "physical train*" OR "exercis* therap*" OR "exercis* movement technique*" OR "physical conditioning"

MeSH:

(MH "Exercise") OR (MH "Plyometric Exercise") OR (MH "Exercise Therapy") OR (MH "Exercise Movement Techniques") OR (MH "Circuit-Based Exercise") OR (MH "Resistance Training") OR (MH "High-Intensity Interval Training") OR (MH "Physical Conditioning, Human") OR (MH "Running") OR (MH "Leisure Activities") OR (MH "Physical Exertion") OR (MH "Physical Fitness") OR (MH "Cardiorespiratory Fitness") OR (MH "Sports")

**Outcome**

Key Words:

(infant* OR baby OR babies OR neonat* OR neo-nat* OR newborn* OR new-born* OR "new born*" OR fetal* OR foetal* OR fetus* OR foetus* OR birth*) ADJ3 (weigh* OR size OR "growth retardation" OR prematur* OR "low birth weigh*" OR hypoxi*)

+ OR birthweight OR prematur* OR "placenta* circulation"

MeSH:

(MH "Birth Weight") OR (MH "Infant, Low Birth Weight") OR (MH "Infant, Very Low Birth Weight") OR (MH "Infant, Extremely Low Birth Weight") OR (MH "Fetal Weight") OR (MH "Infant, Small for Gestational Age") OR (MH "Infant, Premature") OR (MH "Placental Circulation") OR (MH "Fetal Growth Retardation") OR (MH "Fetal Hypoxia") OR (MH "Parity") OR (MH "Pregnancy Outcome") OR (MH "Premature Birth") OR (MH "Fetal Development")

**Cochrane**

**Population**

Key Words:

pregnan* OR gestation* OR trimester* OR ''expect* mother'' OR ''expect* woman*'' OR ''expect* women*'' OR ''prenatal care''

MeSH:

[Pregnant Women] OR [Pregnancy] OR [Pregnancy Trimesters] OR [Pregnancy Trimester, First] OR [Pregnancy Trimester, Second] OR [Pregnancy Trimester, Third] OR [Prenatal Care]

**Intervention**

Key Words:

exercise* OR exercising OR "physical activity" OR "physical activities" OR "physical exert*" OR "physical fitness" OR "fitness train*" OR running OR sport* OR "physical train*" OR "exercis* therap*" OR "exercis* movement technique*" OR "physical conditioning"

MeSH:

[Exercise] OR [Exercise Therapy] OR [Exercise Movement Techniques] OR [Plyometric Exercise] OR [Circuit-Based Exercise] OR [Resistance Training] OR [High-Intensity Interval Training] OR [Physical Conditioning, Human] OR [Running] OR [Leisure Activities] OR [Physical Exertion] OR [Physical Fitness] OR [Cardiorespiratory Fitness] OR [Sports]

**Outcome**

Key Words:

(infant* OR baby OR babies OR neonat* OR neo-nat* OR newborn* OR new-born* OR 'new born*' OR fetal* OR foetal* OR fetus* OR foetus* OR birth*) NEAR/3 (weigh* OR size OR "growth retardation" OR prematur* OR "low birth weigh*" OR hypoxi*)

+ OR birthweight OR prematur* OR "placenta* circulation"

MeSH:

[Birth Weight] OR [Infant, Low Birth Weight] OR [Infant, Very Low Birth Weight] OR [Infant, Extremely Low Birth Weight] OR [Fetal Weight] OR [Infant, Small for Gestational Age] OR [Infant, Premature] OR [Placental Circulation] OR [Fetal Development] OR [Parity] OR [Fetal Hypoxia] OR [Pregnancy Outcome] OR [Premature Birth]

**Web of Science**

**Population**

Key Words:

pregnan* OR gestation* OR trimester* OR ''expect* mother'' OR ''expect* woman*'' OR ''expect* women*'' OR ''prenatal care''

**Intervention**

Key Words:

exercise* OR exercising OR "physical activity" OR "physical activities" OR "physical exert*" OR "physical fitness" OR "fitness train*" OR running OR sport* OR "physical train*" OR "exercis* therap*" OR "exercis* movement technique*" OR "physical conditioning"

**Outcome**

Key Words:

(infant* OR baby OR babies OR neonat* OR neo-nat* OR newborn* OR new-born* OR 'new born*' OR fetal* OR foetal* OR fetus* OR foetus* OR birth*) N3 weigh* OR size OR "growth retardation" OR prematur* OR "low birth weigh*" OR hypoxi*)

+ OR birthweigh* OR prematur* OR "placenta* circulation"

**Embase**

**Population**

Key Words:

pregnan* OR gestation* OR trimester* OR ''expect* mother'' OR ''expect* woman*'' OR ''expect* women*'' OR ''prenatal care''

MeSH:

pregnant woman/ OR pregnancy/ OR first trimester pregnancy/ OR second trimester pregnancy/ OR third trimester pregnancy/ OR expectant mother/ OR prenatal care/

**Intervention**

Key Words:

exercise* OR exercising OR "physical activity" OR "physical activities" OR "physical exert*" OR "physical fitness" OR "fitness train*" OR running OR sport* OR "physical train*" OR "exercis* therap*" OR "exercis* movement technique*" OR "physical conditioning"

MeSH:

exercise/ OR aerobic exercise/ OR anaerobic exercise/ OR circuit training/ OR exercise intensity/ OR high intensity interval training/ OR plyometrics/ OR resistance training/ OR dynamic exercise/ OR physical activity/ OR cycling/ OR running/ OR fitness/ OR kinesiotherapy/ OR movement therapy/ OR muscle training/ OR sport/

**Outcome**

Key Words:

((infant* OR baby OR babies OR neonat* OR neo-nat* OR newborn* OR new-born* OR "new born*" OR fetal* OR foetal* OR fetus* OR foetus* OR birth*) adj3 (weigh* OR size OR "growth retardation" OR prematur* OR "low birth weigh*" OR hypoxi*)) birthweight OR prematur* OR "placenta* circulation"

MeSH:

birth weight/ OR low birth weight/ OR small for date infant/ OR very low birth weight/ OR extremely low birth weight/ OR fetus weight/ OR fetus circulation/ OR fetus hypoxia/ OR intrauterine growth retardation/ OR prematurity/ OR fetus development/ OR parity/ OR placenta circulation/
