# Supplementary figures and images for: The effects of vigorous intensity exercise in the third trimester of pregnancy: a systematic review and meta-analysis
Source: BMC Pregnancy Childbirth. 2019 Aug 7;19:281. doi: 10.1186/s12884-019-2441-1 (PMC6686535; doi:10.1186/s12884-019-2441-1)

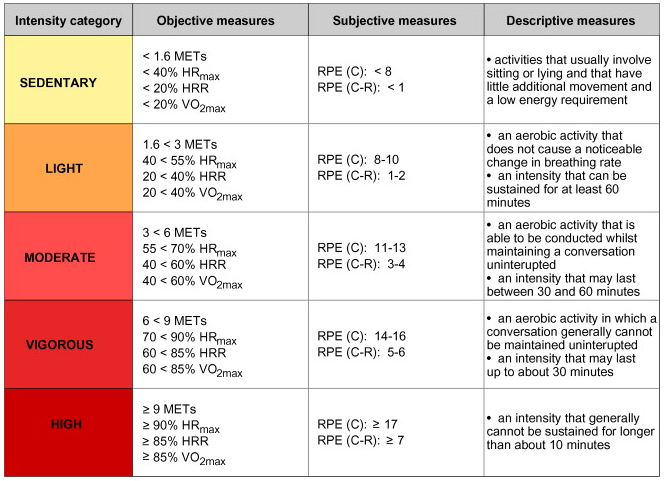

Supplement: Supplementary file 2 — Exercise and Sports Science Australia position statement on physical activity and exercise intensity terminology. Reproduced with permission from Norton et al. [15]. (PNG 151 kb) [file 12884_2019_2441_MOESM2_ESM.png]
