# Supplementary material for: The effects of vigorous intensity exercise in the third trimester of pregnancy: a systematic review and meta-analysis
Source: BMC Pregnancy Childbirth. 2019 Aug 7;19:281. doi: 10.1186/s12884-019-2441-1 (PMC6686535; doi:10.1186/s12884-019-2441-1)
Supplement: Supplementary file 3 — Results from all overall and moderation meta-analyses. (DOCX 15 kb) [file 12884_2019_2441_MOESM3_ESM.docx]

Additional file 3. Results from all overall and moderation meta-analyses.

| Moderator | Mean difference [95% CI] | Hedges' g [95% CI] | p | k | n |
| --- | --- | --- | --- | --- | --- |
| *Birthweight (g)* |  |  |  |  |  |
| Overall | 8.06 [ -57.44, 73.55] | 0.01 [ -0.12, 0.15] | 0.8 | 12 | 8006 |
| Light or less | -0.86 [ -73.59, 71.88] | -0.01 [ -0.16, 0.15] | 1 | 10 | 7734 |
| Moderate or less | 66.83 [ -220, 353.67] | 0.13 [ -0.44, 0.71] | 0.4 | 3 | 272 |
| Retrospective | 62.05 [ -2084.96, 2209.07] | 0.12 [ -4.48, 4.71] | 0.8 | 2 | 158 |
| RCT | -75.15 [ -169.63, 19.33] | -0.18 [ -0.37, 0.02] | 0.1 | 6 | 495 |
| Prospective | 33.6 [ -53.69, 120.89] | 0.07 [ -0.11, 0.24] | 0.4 | 5 | 7353 |
| *Gestational age at delivery (weeks)* | |  |  |  |  |
| Overall | 0.21 [ 0.15, 0.27] | 0.15 [ 0.1, 0.2] | 0 | 7 | 4281 |
| Light or less | 0.21 [ 0.15, 0.27] | 0.15 [ 0.1, 0.2] | 0 | 7 | 4281 |
| Retrospective | 0.18 [ -0.12, 0.48] | 0.14 [ -0.01, 0.29] | 0.1 | 2 | 1767 |
| RCT | 0.16 [ -0.12, 0.44] | 0.12 [ -0.08, 0.33] | 0.2 | 4 | 443 |
| Prospective | 0.26 [ -0.25, 0.78] | 0.16 [ -0.5, 0.81] | 0.1 | 2 | 2071 |
| *Risk of Low birth weight* | |  |  |  |  |
| Overall | 0.44 [ -0.83, 1.7] | 0.25 [ -0.5, 1] | 0.4 | 4 | 2454 |
| Light or less | 0.18 [ -1.17, 1.53] | 0.1 [ -0.68, 0.88] | 0.6 | 3 | 2353 |
| RCT | 0.63 [ -1.45, 2.71] | 0.37 [ -0.82, 1.56] | 0.2 | 2 | 123 |
| Prospective | 0.44 [ -9.58, 10.45] | 0.26 [ -5.55, 6.06] | 0.7 | 2 | 2331 |
| *Maternal weight gain (kg)* | |  |  |  |  |
| Overall | -0.46 [ -2.05, 1.12] | -0.13 [ -0.47, 0.21] | 0.5 | 7 | 1834 |
| Light or less | -0.34 [ -2.19, 1.52] | -0.11 [ -0.52, 0.3] | 0.7 | 6 | 1747 |
| Retrospective | -2.75 [ -13.86, 8.35] | -0.45 [ -3.11, 2.22] | 0.2 | 2 | 158 |
| RCT | -0.45 [ -2.65, 1.76] | -0.1 [ -0.63, 0.43] | 0.6 | 5 | 450 |
| *Risk of Prematurity* | |  |  |  |  |
| Overall | -0.2 [ -0.36, -0.03] | -0.12 [ -0.21, -0.02] | 0 | 4 | 3025 |
| Light or less | -0.16 [ -0.32, 0.01] | -0.09 [ -0.2, 0.01] | 0.1 | 3 | 1644 |
| RCT | -0.41 [ -1.64, 0.82] | -0.24 [ -0.89, 0.41] | 0.2 | 2 | 312 |
| *Risk of Small for gestational age* | |  |  |  |  |
| Overall | 0.15 [ -0.06, 0.35] | 0.1 [ -0.03, 0.23] | 0.1 | 7 | 4504 |
| Light or less | 0.15 [ -0.06, 0.35] | 0.1 [ -0.03, 0.23] | 0.1 | 7 | 4504 |
| RCT | -0.27 [ -1.28, 0.74] | -0.16 [ -0.84, 0.53] | 0.5 | 4 | 451 |
| Prospective | 0.19 [ -0.3, 0.68] | 0.12 [ -0.26, 0.5] | 0.1 | 2 | 3982 |
